# Development and validation of Tanzania’s food literacy tool for adults: implications for healthy eating behaviours

**DOI:** 10.1017/S0007114525105138

**Published:** 2025-09-28

**Authors:** Victoria Kariathi, Hadijah Mbwana, Constance Rybak, Safiness Msollo

**Affiliations:** 1 Sokoine University of Agriculture, Department of Human Nutrition and Consumer Sciences, P.O. Box 3006, Morogoro, Tanzania; 2 Tanzania Food and Nutrition Centre, Department of Nutrition Education and Training, P.O. Box 977, Dar es Salaam, Tanzania; 3 Division Urban Plant Ecophysiology, Faculty of Life Sciences, Thaer-Institute of Agricultural and Horticultural Sciences, Humboldt-Universität zu Berlin, Lentzeallee 55, 14195 Berlin, Germany

**Keywords:** Adults, Food, Food literacy, Nutrition information, Tanzania, Validity

## Abstract

The study aimed to develop and validate a food literacy tool for Tanzanian adults. The Tanzanian nutrition, food and health promotion experts evaluated the initial twenty-three-question food literacy tool for its relevance to the context, where its content validity was determined. The construct validity involved the analysis of food literacy information collected in a cross-sectional study involving 709 adults (484 females and 225 males) sampled from rural and urban Tanzania. Exploratory factor analysis was conducted to explore the underlying factor structure and identify the number of latent constructs. A confirmatory factor analysis using structural equation modelling verified the measurement model and confirmed the theoretical model’s validity and reliability. The descriptive statistics summarised the essential characteristics of the study sample. The final tool remained with fourteen questions after removing questions with low factor loadings < 0·5 and higher uniqueness above 0·60. The model achieved construct validity through convergent and discriminant validity and construct reliability through the composite reliability exceeding 0·60 and a Cronbach’s *α* value of 0·83 and above. The fourteen-question food literacy tool has been reviewed and evaluated by experts in food, nutrition and public health; therefore, it is a valid measure of food literacy among adults in Tanzania. It is suitable for designing nutrition education programmes and ensures accurate and reliable measurements for effective interventions and policy actions.

The triple burden of malnutrition, which is the coexistence of undernutrition, micronutrient deficiency and overweight and obesity^(1)^, affecting both children and adults in Tanzania, poses a significant public health concern^(2,3)^. Malnutrition negatively affects economic growth by lowering productivity and also weakens the immune system, which increases susceptibility to diseases^(4)^. Malnutrition effects are more pronounced in populations within low- and middle-income countries, where limited access to nutritious food, healthcare and sanitation exacerbates the situation^(1,4)^. The impacts of malnutrition are life-threatening and persist at the individual, family, community and national levels^(1,5)^. The Tanzanian government continued to address malnutrition through strategic plans, policies and targeted interventions^(2)^. Some efforts have focused on empowering communities through nutrition training programmes and promotion led by healthcare providers and community health workers^(6)^. As part of these initiatives, nutrition education programmes such as the Integrated Management of Acute Malnutrition, Maternal, Infant, Young Child and Adolescent Nutrition and the National Nutrition Social and Behaviour Change kit were designed to capacitate health care workers and community health workers to deliver nutrition information to the community^(6)^. These nutrition educational programmes target the most vulnerable populations, primarily focusing on preventing and treating childhood undernutrition and reducing maternal anaemia.

Despite the importance of these initiatives for improving the nutrition status of both children and women of reproductive age, there is a lack of targeted nutrition education programmes for adults, especially those aimed at addressing the current increase in overweight and obesity^(6)^. It has been established that the rise in overweight and obesity in the country results from insufficient nutrition education and a lack of awareness about healthy eating practices^(7,8)^. Recently, the government launched the food-based dietary guidelines to guide Tanzanians to adopt healthier eating habits and lifestyle choices for improved nutrition outcomes. The food-based dietary guidelines set a standard for practical, evidence-based recommendations not only for healthy eating and lifestyle behaviours but also for promoting overall health and preventing diet-related NCD^(2)^. Effective utilisation of these nutrition recommendations requires individuals to possess the necessary knowledge, skills and competencies to understand and apply them to make informed food choices. An individual’s ability to access, understand and apply food-related information, knowledge, skills, behaviours and self-efficacy essential for adhering to the recommended dietary intake is referred to as food literacy^(9–11)^. Food literacy has recently been acknowledged as a key determinant of health and well-being^(12)^ due to its potential to promote healthy eating habits and prevent NCD such as obesity, diabetes and CVD^(13)^. Food literacy is essential since it emphasises the linkage between acquiring basic nutritional information and using foods to meet daily dietary needs^(13)^. Studies reported that individuals with sufficient food literacy can translate nutrition information into informed dietary choices to improve their nutritional status and health outcomes^(14,15)^. This signifies that transitioning towards healthy dietary recommendations requires the practical adaptation of food literacy.

Regardless of the need to enhance food literacy, there is a gap in the availability of validated tools for assessing food literacy among the Tanzanian population, particularly adults. While numerous reliable and valid tools exist in other countries and contexts, adaptation and validation of these tools for specific settings are crucial to ensure their relevance and effectiveness in addressing precise needs and challenges^(16,17)^. Tanzania’s diverse culture in rural and urban settings requires a context-specific food literacy tool to address disparities and inform interventions. Recently, Yiga *et al.* ^(16)^ developed and validated a food literacy tool for adults in East Africa, focusing on broader aspects of food systems and information evaluation. However, the tool was designed for urban populations in Kenya and Uganda and has not been tested in Tanzania, where different contextual factors, particularly regarding access to and evaluation of nutrition information, might limit its effectiveness. Furthermore, Conti *et al.* ^(18)^ developed and validated a food knowledge tool for women of reproductive age in Tanzania; nevertheless, it lacks generalisability to the entire adult population as it only targets women of reproductive age. Moreover, since the tool was designed for general dietary habits, it didn’t address the underlying knowledge, skills and competencies that influence food choices. This creates the need for a tool that explicitly measures food literacy to promote sustainable, healthy eating behaviours. Therefore, this study aimed to develop and validate a context-specific assessment tool for measuring the information-related dimensions of food literacy among adults in rural and urban settings in Tanzania. Having a validated tool will facilitate accurate assessment of food literacy and guide interventions to improve dietary behaviours for better health outcomes.

## Methodology

### Process of development and validation of the food literacy tool

**Fig. 1.**
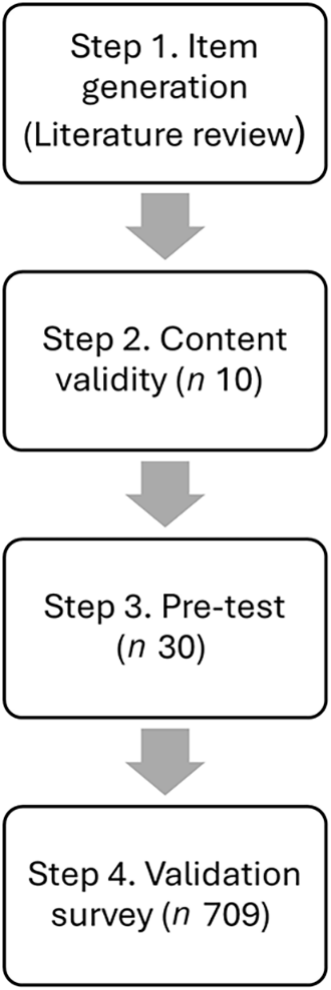
A flow chart representing the process of developing and validating the food literacy tool.

The food literacy tool was developed and validated through four steps: item generation, content validation, pre-tests and validation surveys, as illustrated in Fig. 1.

#### Step 1: Item generation

To generate food literacy questions, a thorough literature review was conducted to identify existing validated food and nutrition literacy questionnaires linked with health literacy. The literature review followed the Preferred Reporting Items for Systematic Reviews and Meta-Analyses guidelines to gather the necessary items^(19)^. A systematic search of the Cochrane Database, Google Scholar and PubMed Central was carried out using Boolean search terms ‘food literacy questionnaire’ AND ‘health literacy’ AND (‘Healthy Plate’ OR ‘Healthy Eating’) AND ‘validation’ AND ‘adults’ AND ‘food literacy’ AND ‘construct validity’ AND ‘nutrition’ AND ‘information’ from the years 2015 to 2023. The same search terms were applied across all databases, and publications were included if (i) they were original research articles featuring a method or instrument to measure food or nutrition literacy, (ii) they evaluated an adult population and (iii) they were written in English. Studies were excluded if they included tools that were (i) direct translations of the original version, (ii) published in languages other than English due to language barriers or (iii) designed for age groups other than adults, such as children, students and adolescents.

#### Step 2: Content validity

A panel of nutrition, food and health promotion experts (*n* 10) evaluated the necessity of the questions and the degree to which the questions contribute to the tool’s purpose. The selected ten experts fall within the acceptable range recommended for the content validation process^(20)^. Subject matter expertise is essential in the content validation process^(21)^; hence, this study randomly selected experts based on their expertise in implementing national and subnational nutrition activities, being members of the Scaling Up Nutrition platform with assigned roles in implementing the second National Multisectoral Nutrition Action Plan II^(6)^. The experts were from the Ministry of Health (*n* 2); the President’s Office – Regional Administration and Local Government, Tanzania (*n* 1); and research institutions represented by the Tanzania Food and Nutrition Centre (*n* 2). Additional experts were from Regional and District Administrative Secretariats (*n* 2); academic institutions, including Sokoine University of Agriculture (*n* 1) and the Nelson Mandela African Institution of Science and Technology (*n* 1); and UN agencies (*n* 1). The online semi-structured questionnaire was sent to these experts, who evaluated every question by answering each food literacy question to determine its necessity and relevance for measuring food literacy among adults in Tanzania. Using the information provided by the subject matter experts, the calculation of the content validity ratio (CVR), content validity index (CVI), scale validity index (S-CVI) and experts in agreement was done to ensure that only accepted questions remain in the tool.

##### Content validity ratio

The CVR was calculated to determine whether a question should be included in the tool. The experts rated each question using a 3-point Likert scale: 1 = not necessary; 2 = useful but not necessary; and 3 = essential. For each question, CVR was calculated using the formula provided in equation 1 ^(21,22)^.
(1)

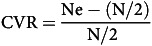




where:

Ne = number of experts selected essential

N = total number of evaluators

For ten experts, the minimum acceptable CVR level is 0·62^(21)^.

##### Content validity index

The experts evaluated each question using a 4-point Likert scale from (1) ‘not relevant’, (2) ‘somewhat relevant’, (3) ‘relevant’ to (4) ‘highly relevant’ in relation to the food literacy concept. This study calculated the individual content validity index (I-CVI) and the S-CVI. The I-CVI was determined based on the proportion of experts scoring a question with a 3 or 4 using equation 2 ^(21)^. If the value of I-CVI is greater than 0·78, then the question remains.
(2)

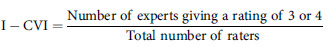




The S-CVI based on the averaging method (AVE) was calculated by summing all I-CVI scores and dividing by the total number of questions using equation 3.
(3)

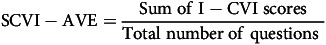




According to Madadizadeh and Bahariniya^(21)^, the minimum S-CVI of 0·90 was considered adequate

##### Expert in agreement

This was calculated by summing the number of experts who scored 3 or 4, indicating their agreement with each food literacy question. If the number of experts in the agreement exceeds half for each question, then the question remains in the tool for further analysis^(20)^.

#### Step 3: Pre-test

To evaluate the readability, feasibility and consistency of the pre-designed food literacy tool, a pre-test was conducted with thirty adults (fifteen males and fifteen females), which is the recommended sample size for pre-testing a psychometric tool^(23)^. Participants were recruited through the local authorities’ channels in the Morogoro region. The trained enumerators clarified each question of the food literacy tool while recording participants’ responses. The pre-test aided in optimising the tool and enhancing a clear understanding of each question.

#### Step 4: Validation survey

A validation survey was carried out from February to March 2023 to collect information on food literacy, which was necessary for evaluating the construct validity and reliability of the food literacy tool. The methodology used in the validation survey is detailed below.

##### Sampling methods

The study is part of the FoCo-Active project, ‘Addressing the triple burden of malnutrition through the behavioural change in food consumption and physical activity: A rural-urban comparative study’. The project is implemented in the Ilala and Mkuranga districts of Dar es Salaam and the Pwani regions of Tanzania, respectively. The study employed a school-based approach and a multistage cluster sampling method with stratification to select a sample of school children from both rural and urban areas. The sample size for school children was determined using Yamane’s formula as indicated in equation 4 ^(24)^.
(4)

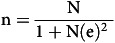




where:

n = the desired sample size

N = the target population size

e = the percentage level of precision^(24)^

The initial stage in this multistage sampling involved the purposive selection of two study sites –Ilala (an urban setting) in Dar es Salaam and Mkuranga (a rural setting) in the Pwani region – as the primary sampling units. In the second stage, a purposive selection of wards was made from each selected district. Two wards, namely, Kisegese and Mkamba, were chosen from the Mkuranga, and three wards – Gongolamboto, Upanga and Kinyerezi – were chosen from Ilala. The ward selection in Ilala was designed to ensure adequate representation of low, middle- and high-socio-economic status (SES) groups based on geographical locations of residency, whereas in Mkuranga, the focus was on selecting wards that characterise rural Tanzanian farming communities. The third stage involved randomly selecting primary schools from each chosen ward: two schools per ward in Ilala and one per ward in Mkuranga. Two schools were selected from Mkuranga and six from Ilala, proportional to population sizes. In the fourth stage, a random sampling of pupils from standard one to four from the sampled schools was conducted. The parent(s) or adult caretaker(s), male and/or female, living in the same household as the sampled child, participated in this study.

##### Inclusion and exclusion criteria

The participants were included if they were 18 years or older, representing one male and/or female residing in the same household as the selected child. Hence, of the 714 adults interviewed, five participants were excluded because either another male or female adult in the same household had been interviewed or was under 18 years of age. Therefore, the analysis involved 709 participants (484 females and 225 males). Among these, 495 were from the Ilala district, and 194 were from the Mkuranga district. The sample size allocation in each study site was proportional to the area’s population size. The total sample size met the recommended guideline for factor analysis^(25)^.

##### Description of the study area

The selected study areas have tropical climatic conditions and are close to the equator and the warm Indian Ocean; hence, they experience humid and hot weather throughout the year. According to the National Census of 2022, the population size of Mkuranga was 533 033, while that of Ilala was 1 649 912^(26,27)^. The total area for the Mkuranga district is 2827 km^2^, and Ilala is 364·9 km^2^. The selected areas have two rainy seasons: a short rainy season from November to December and a longer one from March to June. The main activities in the Mkuranga district are livestock keeping and crop farming, especially cassava, fruits, vegetables and coconuts. The main economic activities in Ilala include trading, transportation services, agriculture, medical, handicrafts, banking and construction. Other economic activities are vegetable production and small and medium-sized industries that process beverages and foods^(27)^.

### Statistical analysis

Statistical analysis for this study was performed using Microsoft Excel and Stata software version 15.0. Descriptive statistics were carried out to summarise the participants’ basic characteristics. Frequencies, means, sd and percentages were obtained for demographic information of the study participants. Microsoft Excel computed the level of experts’ agreement and content validity. Construct validity and reliability were determined using Stata software. The SES index was established using principal component analysis, which reduced variables and aligned them based on their interrelationships. Key variables included for investigating the SES index were monthly household total expenditure, weekly household food expenditure, median income, toilet status, formal employment, self-employment, literacy level, food poverty, water source, having a refrigerator, household head education, household head employment status, number of illiterate adults and number of illiterate children. The overall Kaiser–Meyer–Olkin value of 0·7265 shows the dataset is suitable for principal component analysis, with all variables having acceptable values above 0·5, indicating sufficient shared variance. Moreover, the variance inflation factor results showed that multicollinearity was not a significant issue among the variables^(28)^.

#### Construct validity and reliability of the measured model

Exploratory factor analysis was conducted to explore the underlying factor structure of the measured variables and identify the number of latent constructs represented by the observed items. This process aimed to select an appropriate number of important factors to explain all essential relationships among variables. Subsequently, a multivariate confirmatory factor analysis was performed using structural equation modelling to verify the measurement model relationship and ensure that the proposed theoretical model is valid and reliable. Structural equation modelling is a statistical model that combines factors and paths to represent the hypothesised relationship between observed indicators and latent constructs^(29)^. Model fit was evaluated using standard goodness-of-fit indices. The factor analysis’s sampling adequacy was determined using a Kaiser–Meyer–Olkin value. A Kaiser–Meyer–Olkin value greater than 0·60 at *P* < 0·05 is appropriate for factor analysis^(30)^.

##### Construct validity

Construct validity assesses the relationship between indicators in the tool and the latent variables. The construct validity of the measured model was assessed by convergent and discriminant validity. Convergent validity was established by calculating the average variance extracted (AVE), while the square root of AVE was used to calculate discriminant validity in comparison with the correlation coefficient between the constructs. AVE indicates the extent to which the indicator variance can be explained by the latent variable. In contrast, discriminant validity shows the extent to which indicators of various latent variables are not related^(31)^. The AVE of ≥ 0·5 and the discriminant validity value exceeding that of the correlation coefficient of the latent variables prove the validity of each construct^(32)^.

##### Construct reliability

Construct reliability (CR) refers to the extent to which an instrument measures what it is intended to measure^(25)^. The reliability of this study was assessed using Cronbach’s *α* and composite reliability. The Cronbach’s *α* was obtained through factor analysis, while the CR was calculated based on standardised factor loadings using equation 5.
(5)

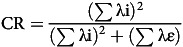




where λ = standardised factor loading to question i and ε = error of variance for question i. The error of variance (ε) is estimated using equation 6.
(6)

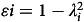




A Cronbach’s *α* score above 0·7 and a CR above 0·6 indicate the internal consistency of a scale within the multidimensional food literacy construct, serving as an indicator of reliability^(25)^.

## Results

### Item generation

The literature review primarily identified questions regarding food and nutrition information, as well as healthy eating, outlined in various reviewed studies^(17,33,34)^. Fifteen publications were retrieved and evaluated for purpose, scope, face validity, content validity, construct validity and reliability for adoption. Adjustments regarding food groups and recommended portions for fruit and vegetable consumption were made as outlined in the Tanzania food-based dietary guidelines to ensure the final tool comprehensively addresses food literacy that suits the Tanzanian context. Table 1 presents the initial pre-designed food literacy tool, which consists of twenty-three questions in a single factor.

**Table 1.** Pre-selected food literacy questions

SN	Food literacy question	Min-Max
FL01	How well do you understand nutrition information (leaflets, brochures)?	Very hard 1 to very easy 5
FL02	How well do you understand mobile nutrition messages?	Very hard 1 to very easy 5
FL03	How well do you understand TV or radio programmes on nutrition?	Very hard 1 to very easy 5
FL04	How well do you understand nutrition information from social media?	Very hard 1 to very easy 5
FL05	How well do you understand nutrition information from in-person social networks (family, friends, etc.)?	Very hard 1 to very easy 5
FL06	How well do you understand nutrition information from government websites?	Very hard 1 to very easy 5
FL07	How well do you understand nutrition information from entertainment education (music, drama and traditional dances)?	Very hard 1 to very easy 5
FL08	How well do you understand nutrition information from browser internet searches (Google, Bing, etc.)?	Very hard 1 to very easy 5
FL09	How well do you understand oral recommendations regarding nutrition from professionals?	Very hard 1 to very easy 5
FL010	There is a lot of information available on healthy nutrition today. How well do you manage to choose the information relevant to you?	Very hard 1 to very easy 5
FL011	How easily can you judge if media information on nutritional issues can be trusted?	Very hard 1 to very easy 5
FL012	Commercials often relate food to health. How easy is it for you to judge whether the presented associations are appropriate?	Very hard 1 to very easy 5
FL013	How easy can you evaluate if a specific food is relevant for a healthy diet?	Very hard 1 to very easy 5
FL014	How easy is it for you to evaluate the longer-term impact of your dietary habits on your health?	Very hard 1 to very easy 5
FL015	In the past, how often were you able to help your family members or a friend if they had questions concerning nutritional issues?	Never 1 to always 5;
FL016	When I have questions on nutrition, I know where I can find information on this issue.	Disagree strongly 1 to agree strongly 5
FL017	Think about a usual day: how easy or difficult is it to compose a balanced meal at home using six recommended food groups?	Very hard 1 to very easy 5
FL018	How easy is it for you to eat takeaways or fast food outside or at home?	Very hard 1 to very easy 5
FL019	How easy is it for you to modify recipes to make them healthier?	Very hard 1 to very easy 5
FL020	How easy is it for you to buy food in an efficient way that saves money and time?	Very hard 1 to very easy 5
FL021	I know the official Tanzanian recommendations about fruit and vegetable consumption.	Disagree strongly 1 to agree strongly 5
FL022	I am familiar with the Tanzanian-recommended food groups for healthy meals.	Disagree strongly 1 to agree strongly 5
FL023	How easy is it for you to cook meals at home using healthy ingredients?	Very hard 1 to very easy 5

### Content validity

Table 2 shows the results for expert agreements, CVR, I-CVI and S-CVI. More than half of the experts agreed that the questions were necessary to measure food literacy. The CVR from this study was 0·85, the S-CVI was 0·9 and the I-CVI ranged from 0·7 to 1·0, all within acceptable limits. Although the pre-designed food literacy tool met acceptable content validity standards, experts suggested including questions about understanding food labels, efforts to prevent NCD, lifestyle behaviours and health-related decision-making. Six questions presented in Table 3 were adapted from a validated health literacy questionnaire and incorporated into the pre-designed tool for further construct validity and reliability analysis of twenty-nine questions.

**Table 2.** Expert agreement on twenty-three pre-selected FL questions

SN	Food literacy question	Expert in agreement *n* 10	Percent (%)	CVR^*^	I-CVI^†^
FL01	How well do you understand nutrition information (leaflets, brochures)?	9	90	0·8	0·9
FL02	How well do you understand mobile nutrition messages?	8	80	0·6	0·8
FL03	How well do you understand TV or radio programmes on nutrition?	10	100	1	1
FL04	How well do you understand nutrition information from social media?	10	100	1	1
FL05	How well do you understand nutrition information from in-person social networks (family, friends, etc.)?	10	100	1	1
FL06	How well do you understand nutrition information from government websites?	9	100	0·8	0·9
FL07	How well do you understand nutrition information from entertainment education (music, drama and traditional dances)?	8	80	0·6	0·8
FL08	How well do you understand nutrition information from browser internet searches (Google, Bing, etc.)?	9	90	0·8	0·9
FL09	How well do you understand oral recommendations regarding nutrition from professionals?	10	100	1	1
FL010	There is a lot of information available on healthy nutrition today. How well do you manage to choose the information relevant to you?	10	100	1	1
FL011	How easily can you judge if media information on nutritional issues can be trusted?	8	80	0·6	0·8
FL012	Commercials often relate food to health. How easy is it for you to judge whether the presented associations are appropriate?	10	100	1	1
FL013	How easy can you evaluate if a specific food is relevant for a healthy diet?	10	100	1	1
FL014	How easy is it for you to evaluate the longer-term impact of your dietary habits on your health?	8	80	0·6	0·8
FL015	In the past, how often were you able to help your family members or a friend if they had questions concerning nutritional issues?	8	80	0·6	0·8
FL016	When I have questions on nutrition, I know where I can find information on this issue.	9	90	0·8	0·9
FL017	Think about a usual day: how easy or difficult is it to compose a balanced meal at home using six recommended food groups?	7	70	0·8	0·7
FL018	How easy is it for you to eat takeaways or fast food outside or at home?	7	70	1	0·7
FL019	How easy is it for you to modify recipes to make them healthier?	7	70	1·25	0·7
FL020	How easy is it for you to buy food in an efficient way that saves money and time?	7	70	1·25	0·7
FL021	I know the official Tanzanian recommendations about fruit and vegetable consumption.	9	90	0·8	0·9
FL022	I am familiar with the Tanzanian-recommended food groups for healthy meals.	9	90	0·8	0·9
FL023	How easy is it for you to cook meals at home using healthy ingredients?	7	70	0·4	0·7
				0·85	0·87
	S-CVI^‡^				0·90

* CVR, content validity ratio.

† I-CVI, individual content validity index.

‡ S-CVI, scale validity index.

**Table 3.** Additional food literacy questions

SN	Questions	Min-Max
FL024	How easy would you say it is to find information on how to prevent or manage conditions like obesity, high blood pressure or high cholesterol?	Very difficult 1 to very easy 5
FL025	How easy would you say it is to find information on healthy activities such as exercise, healthy food and nutrition?	Very difficult 1 to very easy 5
FL026	How easy would you say it is to find out about efforts to promote your health at work?	Very difficult 1 to very easy 5
FL027	How easy would you say it is to understand information on food packaging?	Very difficult 1 to very easy 5
FL028	How easy would you say it is to make decisions to improve your health?	Very difficult 1 to very easy 5
FL029	How easy would you say it is to take part in activities that improve health and well-being in your community?	Very difficult 1 to very easy 5

### Sample characteristics for construct validity and reliability measurements

Sixty-eight percent of respondents were women, and 72 % resided in urban areas. The average age of respondents was 38 years, ranging from 18 to 80. About 81 % of participants were married, and 55 % had completed primary school education. Approximately 31 % of the participants were classified as high SES, while another 31 % were categorised as low SES, indicating an even distribution between higher and lower economic groups. Further details can be found in Table 4.

**Table 4.** Descriptive characteristics of participants

Characteristics	Frequency (*n* 709)	Percent (%)
Gender		
Female	484	68·3
Male	225	31·7
Residence		
Urban (Ilala)	515	72·6
Rural (Mkuranga)	194	27·4
Age categories		
Below 20	5	0·7
20–24	30	4·2
25–29	103	14·5
30–34	143	20·2
35–39	150	21·2
40–44	111	15·7
45–49	83	11·7
50 and above	84	11·9
Marital status		
Never married	62	8·7
Married or living together	577	81·4
Divorced/separated or widowed	70	9·9
Education attained		
No education	76	10·7
Primary incomplete	45	6·4
Primary complete	390	55·0
Secondary and above	198	27·9
Socioeconomic status		
High	225	31·7
Middle	113	15·9
Low	224	31·6
Not specified	147	20·73

### Construct validity and reliability

#### Factor structure

Factor analysis was done using exploratory factor analysis on the twenty-nine food literacy items. The overall Kaiser–Meyer–Olkin value of 0·94 proves that the observed variables are suitable for measuring sampling adequacy for factor analysis. Three-factor components were extracted as they explain 88·04 % of the variance. According to suggestions from Cheung *et al.* ^(32)^ protocol, fifteen questions were removed due to high uniqueness > 0·60 or low factor loading < 0·50; hence, the study proceeded with fourteen questions. The cut-off points for both factor loading and uniqueness are established by the guidelines in the factor analysis^(31)^. The remaining questions with factor loadings > 0·50 indicate an association with the latent construct and can best explain the constructs and align with pre-established theory to confirm model expectations. A rotational varimax analytical procedure aligned the variables according to the three factors. The sorted factor loadings and uniqueness of the three retained factors are presented in Table 5. The three factors were named as understanding nutrition information, applying nutrition information and healthy eating. The constructs were named based on Nutbeam’s model of health literacy, which differentiates functional, interactive and critical literacy^(35)^. The overall model demonstrates goodness of fit, as evidenced by root mean square error of approximation of 0·078, which falls within the acceptable range of 0·05–1·00, as well as Comparative Fit Index and Tucker–Lewis Index of 0·942 and 0·930, respectively, both exceeding the threshold of 0·90^(31,32,36)^. This model was then tested with confirmatory factor analysis through structural equation modelling, enabling correlations among the three latent factors.

**Table 5.** Sorted factor loadings and uniqueness of the retained variables

SN	Variable	Factor 1	Factor 2	Factor 3	Uniqueness
FL01	How well do you understand nutrition information (leaflets, brochures)?	0·728	0·284	0·289	0·307
FL02	How well do you understand mobile nutrition messages?	0·726	0·228	0·272	0·347
FL03	How well do you understand TV or radio programmes on nutrition?	0·707	0·210	0·235	0·401
FL04	How well do you understand nutrition information from social media?	0·609	0·283	0·261	0·481
FL05	How well do you understand nutrition information from in-person social networks (family, friends, etc.)?	0·662	0·173	0·117	0·518
FL07	How well do you understand nutrition information from entertainment education (music, drama and traditional dances)?	0·610	0·136	0·173	0·580
FL09	How well do you understand oral recommendations regarding nutrition from professionals?	0·646	0·101	0·134	0·555
FL010	There is a lot of information available on healthy nutrition today. How well do you manage to choose the information relevant to you?	0·159	0·710	0·254	0·406
FL011	How easily can you judge if media information on nutritional issues can be trusted?	0·181	0·750	0·244	0·345
FL012	Commercials often relate food to health. How easy is it for you to judge whether the presented associations are appropriate?	0·206	0·754	0·147	0·367
FL013	How easy can you evaluate if a specific food is relevant for a healthy diet?	0·172	0·730	0·270	0·365
FL014	How easy is it for you to evaluate the longer-term impact of your dietary habits on your health?	0·238	0·722	0·170	0·393
FL021	I know the official Tanzanian recommendations about fruit and vegetable consumption.	0·203	0·243	0·854	0·170
FL022	I am familiar with the Tanzanian-recommended food groups for healthy meals.	0·226	0·220	0·855	0·171

#### Internal consistency or reliability

Table 6 shows the measurement model’s results, including both Cronbach’s *α* and CR for each subscale. The overall Cronbach’s *α* value is 0·93, and it is greater than 0·83 for each of the specified latent variables, while the CR ranges from 0·79 to 0·93.

**Table 6.** Results of measurement model (*n* 709)

SN	Measurement	M	sd ^*^	AVE^†^	Cronbach’s *α*	CR^‡^
	Understanding nutrition information					
FL01	How well do you understand nutrition information (leaflets, brochures)?	3·416	1·461			
FL02	How well do you understand mobile nutrition messages?	3·322	1·461			
FL03	How well do you understand TV or radio programmes on nutrition?	3·573	1·421			
FL04	How well do you understand nutrition information from social media?	2·976	1·532			
FL05	How well do you understand nutrition information from in-person social networks (family, friends, etc.)?	3·612	1·446			
FL07	How well do you understand nutrition information from entertainment education (music, drama and traditional dances)?	2·969	1·570			
FL09	How well do you understand oral recommendations regarding nutrition from professionals?	3·810	1·494	0·537	0·889	0·793
	Apply food and nutrition information					
FL010	There is a lot of information available on healthy nutrition today. How well do you manage to choose the information relevant to you?	3·021	1·392			
FL011	How easily can you judge if media information on nutritional issues can be trusted?	2·958	1·441			
FL012	Commercials often relate food to health. How easy is it for you to judge whether the presented associations are appropriate?	2·687	1·429			
FL013	How easy can you evaluate if a specific food is relevant for a healthy diet?	2·911	1·479			
FL014	How easy is it for you to evaluate the longer-term impact of your dietary habits on your health?	2·834	1·464	0·626	0·895	0·840
	Healthy eating					
FL021	I know the official Tanzanian recommendations about fruit and vegetable consumption.	2·646	1·419			
FL022	I am familiar with the Tanzanian-recommended food groups for healthy meals.	2·599	1·454	0·876	0·967	0·925

* sd, standard deviation.

† AVE, average variance extracted.

‡ Construct reliability.

The observed AVE for the latent variables ranges from 0·54 to 0·88, and the discriminant validity exceeds the correlation coefficient of the latent variables, as indicated in Table 7. The results indicate that the tool, containing fourteen food literacy questions organised into three constructs, is valid and reliable for assessing food literacy among adults.

**Table 7.** The discriminant validity index summary

Construct	Understand nutrition information	Apply nutrition information	Healthy eating
Understand nutrition information	**0·733**		
Apply nutrition information	0·567	**0·791**	
Healthy eating	0·555	0·534	**0·936**

Diagonal in bold presents the square root of AVE while off diagonal presents the correlations.

## Discussion

This study aimed to develop and validate a food literacy assessment tool adaptable to the adult population in rural and urban areas of Tanzania. Based on experts’ and analytical evidence, the measures met satisfactory levels, implying the tool’s appropriateness and reliability for measuring food literacy of the target population. Following the results, this discussion provides details of the content validity, construct validity, reliability, domain-specific interpretation, practical and policy implications and the study’s strengths and limitations.

### Content validity

The content validation process with subject matter specialists from a local context is the first and critical step in ensuring the items’ relevance and representativeness of the key construct^(21)^. In Tanzania, engaging key stakeholders from the national to subnational levels in the content validation process, as specified by the National Multisectoral Nutrition Action Plan II coordination structure^(6)^, is the best practice for ensuring diverse expert input and the successful adoption of the developed tool^(37)^. The study’s findings of an average CVR above 0·62 and I-CVI above 0·70 suggest that all proposed questions should be retained in the tool. The S-CVI value of 0·90 indicates that the overall tool met an excellent standard^(21)^ for the ability to measure food literacy. Both the CVR, I-CVI and S-CVI indices, obtained through expert analysis, met the recommended values, confirming the content validity of the developed food literacy measurement tool^(38)^. While these content validity values are acceptable, experts’ recommendations for additional questions underscore the importance of including local subject matter specialists to ensure culturally and contextually relevant inputs. This ensures the tool remains with useful content that is culturally acceptable, extending beyond mere statistical focus measures. This expert’s evaluation confirmed that this food literacy tool is suitable for use in educational and community settings^(16,37)^. Additionally, the content validity results support further statistical analysis to verify the reliability of the tool.

### Construct validity, reliability and domain structure

The exploratory factor analysis started with twenty-nine questions, but fifteen were eliminated because of low factor loadings and high uniqueness. This refinement led to a three-factor structure where items loaded strongly on their respective factors, reflecting distinct yet related constructs^(31)^. This hypothesised factor structure was later tested and verified using confirmatory factor analysis through structural equation modelling, which showed strong model fit indices, supporting the validity of the hypothesised factor model. The reliability of the food literacy constructs was confirmed using both Cronbach’s *α*, which assessed the internal consistency, and CR, which evaluated reliability based on standardised loadings of confirmatory factor analysis^(32,39)^. A Cronbach’s *α* exceeding 0·8 and a CR above 0·6 demonstrate strong internal consistency and reliable construct measurement across the fourteen questions assessing food literacy. The construct validity observed through AVE of 0·5 and above, along with a discriminant validity greater than the correlation coefficient of the latent variables, indicates that the three constructs in the model met the convergent and discriminant validity criteria. The fourteen retained questions from the previously pre-designed food literacy tool represent the key elements that adequately capture the multidimensional nature of food literacy for practical applications^(31)^.

This study utilised most of the questions from the Short Food Literacy Questionnaire, a validated instrument for Swiss adults introduced by Gréa Krause *et al.* ^(17)^, which initially consisted of a single factor. However, during exploratory factor analysis, the questions were arranged into three-factor components: understanding nutrition information, applying nutrition information and healthy eating. The naming of the constructs considered the domains of food literacy derived from Nutbeam’s model of health literacy, which distinguishes functional, interactive and critical literacy^(35)^. Specifically, ‘understanding nutrition information’ corresponds to functional literacy, reflecting basic comprehension of food and nutrition information; ‘apply’ aligns with interactive literacy, encompassing the skills to use information in daily activities; and ‘health eating’ integrates elements of both interactive and critical literacy by capturing the ability to make informed choices and engage in health-promoting eating behaviours^(17,40)^. This domain-specific placement of questions may reflect cultural, educational or dietary differences that shape how individuals understand and apply food literacy, underscoring the need for localised validation instead of assuming a universal structure. The results are consistent with Zwierczyk *et al.* ^(41)^, who found a three-factor structure: ‘information accessing’, ‘knowledge’ and ‘information appraisal’ when validating the Swiss Short Food Literacy Questionnaire for Poland’s adult population. Contrary to the current findings, Durmus *et al.* ^(42)^ confirmed the unidimensional structure for the Turkish Short Food Literacy Questionnaire, as identified by Gréa Krause *et al.* ^(17)^. Additionally, some questions from the original Swiss Short Food Literacy Questionnaire^(17)^ did not meet the validity criteria in the present study due to contextual irrelevance and cultural differences. This highlights the significance of validating food literacy tools to ensure their relevance, accuracy and effectiveness for specific populations.

### Domain-specific interpretation

Interestingly, having a similar structure and questions does not guarantee that particular questions will appear in the same domain, especially when applied in a different context^(33)^. This observation is made by the tool presented by Zwierczyk *et al.* ^(41)^, even though many questions are similar to the present tool; for example, ‘*There is a lot of information available on healthy nutrition today. How well do you manage to choose the information relevant to you?*’ was classified differently in the two tools. In this study, it falls under the application of the nutrition information domain, whereas Zwierczyk *et al.* ^(41)^ categorised it under the information access domain. This suggests that the interpretation of food literacy questions varies based on how individuals conceptualise information, influenced by cultural, geographical and demographic factors. Furthermore, to inform future interventions, it is essential to understand the details of the questions retained in each domain to illustrate how each domain is represented. The general look of three domains demonstrates how individuals engage with, understand and utilise nutritional information to make healthier dietary choices. Based on the retained questions, improving individual food literacy in this context requires three key aspects: (i) use of both interpersonal and other media platforms information, (ii) equipping individuals with the competence to evaluate the reliability of nutrition information sources critically and (iii) prioritising healthy eating information as recommended by the guidelines. This domain-specific interpretation supports the instrument’s applicability to the local setting, especially when designing future food literacy interventions.

### Practical and policy implications

This is the first food literacy tool validated to assess adult food literacy in Tanzania. The robustness of this measurement tool underscores its ability to accurately contribute to measuring food literacy among adults, specifically in the selected areas. This validated tool provides valuable opportunities to inform policy and public health initiatives. In public health initiatives, it will help in identifying knowledge gaps in comprehending food and nutrition information prior to designing targeted nutrition education programmes and campaigns^(34)^. This will ensure appropriate materials are designed to inform dietary behaviour changes. While many targeted nutritional education programmes exist nationwide, incorporating this tool into current national initiatives will ensure that the delivered information effectively promotes healthy eating habits, hence reducing the burden of malnutrition. Policymakers can incorporate it into national nutrition surveillance systems to monitor progress towards dietary guidelines and non-communicable disease (NCD) prevention goals. Additionally, the tool supports the evaluation of community-based interventions and helps allocate resources by identifying priority areas for improving food literacy and dietary habits. Effective utilisation of the tool in designing interventions will impart confidence and behaviours supporting improved diet quality, preventing NCD and ultimately enhancing an individual’s health outcomes^(41,43)^.

### Strengths and limitations

The strength of this tool comes from its expert validity, a large sample size for reliability testing, a diverse group of adults across different ages and educational backgrounds and its applicability in both rural and urban settings. It is short and easily understandable, with proven validity and reliability. Context specificity and expert validity of this tool enhance its applicability in different settings, making it effective for both rural and urban areas of Tanzania. Despite its strength, this tool has several limitations. It might not fully address the specific needs of different people in other contexts or populations not covered in this study without adaptation or translation. In other regions and low- and middle-income countries settings, researchers can modify culturally specific food groups, language and contextual factors while maintaining the core constructs of food literacy to ensure relevance and validity. Furthermore, the tool has been designed to be brief and easily understood; hence, it might limit the depth of information collected, affecting the assessment’s comprehensiveness.

### Conclusion and recommendations

This expert-based food literacy tool, comprised of fourteen questions, represents a significant advancement in measuring food literacy among adults across diverse educational levels, age groups and settings. The tool can be used in various studies and assessments of food and nutrition literacy interventions. Future research should enhance its sensitivity by exploring its suitability for specific age groups, such as school-age children and adolescents. To enhance its broader relevance, pilot studies should be conducted to assess the feasibility and applicability of this tool in diverse population settings.
